# Epicardial Adipose Tissue and Liver Fibrosis in Patients With Type 2 Diabetes Mellitus and Metabolic Dysfunction-Associated Liver Disease

**DOI:** 10.31083/RCM39534

**Published:** 2025-08-28

**Authors:** Simona Cernea, Andrada Larisa Roiban, Danusia Onișor, Nora Rat

**Affiliations:** ^1^Department M3/Internal Medicine I, George Emil Palade University of Medicine, Pharmacy, Science, and Technology of Târgu Mureş, 540142 Târgu Mureş, Romania; ^2^Diabetes, Nutrition and Metabolic Diseases Outpatient Unit, Emergency County Clinical Hospital, 540136 Târgu Mureş, Romania; ^3^Diabetes Compartment, Mediaș Municipal Hospital, 551030 Mediaș, Romania; ^4^Doctoral School of Medicine and Pharmacy, George Emil Palade University of Medicine, Pharmacy, Science, and Technology of Târgu Mureş, 540142 Târgu Mureş, Romania; ^5^Department ME2/Internal Medicine VII, George Emil Palade University of Medicine, Pharmacy, Science and Technology of Târgu Mureş, 540142 Târgu Mureş, Romania; ^6^Gastroenterology Clinic, Mureș County Clinical Hospital, 540072 Târgu Mureş, Romania; ^7^Department M3/Internal Medicine VI, George Emil Palade University of Medicine, Pharmacy, Science, and Technology of Târgu Mureş, 540142 Târgu Mureş, Romania; ^8^Department of Cardiology, Emergency County Clinical Hospital, 540136 Târgu Mureş, Romania

**Keywords:** epicardial adipose tissue, T2DM, MASLD, liver fibrosis

## Abstract

**Background:**

Epicardial adipose tissue (EAT) is an indicator of high cardiovascular and metabolic risk. This study aimed to investigate the association between EAT thickness (EATT) and liver fibrosis and steatosis in patients with type 2 diabetes mellitus (T2DM) and metabolic dysfunction-associated steatotic liver disease (MASLD).

**Methods:**

Patients with T2DM and MASLD underwent a complex evaluation, which included clinical, laboratory, and liver and transthoracic cardiac ultrasound assessments. The EATT was measured using the standard method. Liver fibrosis and steatosis were evaluated by several non-invasive indexes, through which patients with severe steatosis and advanced fibrosis were identified. Correlations between the EATT and markers of liver fibrosis and steatosis were evaluated by bivariate and multiple regression analyses.

**Results:**

In this study population of 267 T2DM patients with MASLD, the median EATT value was 7 mm. 43.8% of study patients had an EATT >7 mm. The EATT was higher in patients with advanced liver fibrosis (8.97 ± 2.88 mm vs. 7.09 ± 2.38 mm; *p* < 0.0001) and in those with more severe hepatic steatosis (7.69 ± 2.70 mm vs. 6.61 ± 1.88 mm; *p* = 0.0310). A higher percentage of patients with advanced liver fibrosis had an EATT of >7 mm (68.3% vs. 36.7%; odds ratio (OR) = 3.72 [95% confidence interval (CI): 2.02; 6.87]; *p* < 0.0001). In the bivariate analyses, the EATT significantly correlated with the markers of body adiposity, non-invasive indexes of liver steatosis and fibrosis, aspartate aminotransferase (ASAT), gamma glutamyl transpeptidase (GGT), diabetes duration, and pO2. The multiple regression analyses indicated that the EATT was independently associated with fibrosis-4 (FIB-4) score and body fat mass, and with serum ferritin (in fully adjusted models), while the correlation with the markers of hepatic steatosis became non-significant after adjustments for body adiposity.

**Conclusion:**

T2DM patients with MASLD and markers of advanced liver fibrosis have higher EATT, which was independently associated with liver fibrosis.

## 1. Introduction

Epicardial adipose tissue (EAT) is considered a unique visceral fat depot due to its unobstructed proximity to the myocardium and its specific transcriptome and secretome profile, which can become harmful and play a role in the pathogenesis of coronary heart disease (CHD), atrial fibrillation, or heart failure (HF) (mainly with preserved ejection fraction (EF)) [1]. The mechanisms by which EAT contributes to the pathogenesis of heart diseases are complex and include inflammation and increased secretion of pro-inflammatory adipokines, infiltration of free fatty acids and lipotoxicity, adipocyte stress, insulin resistance, release of profibrotic factors, etc., [1, 2]. In patients with diabetes mellitus (DM), hyperglycemia-associated upregulation of signaling through the binding of the advanced glycation end products to their receptors further contribute to these mechanisms by increasing oxidative stress and causing endothelial damage [1, 3].

The EAT volume and thickness were shown to be greater in subjects with CHD and in those with type 2 diabetes mellitus (T2DM) [4, 5, 6, 7]. In a single center study of 142 patients with T2DM, the ultrasound measured EAT thickness (EATT) predicted incident coronary artery disease better than other traditional risk factors [8]. Furthermore, the study by Opincariu D *et al*. [6] demonstrated that in patients with T2DM and acute myocardial infarction, the EATT was linked to significant ongoing inflammation and worse long-term outcomes, evidenced by lower EF, enlargement of the ventricular cavities, and development of ventricular remodeling.

In addition, enlarged EAT is also associated with the metabolic syndrome and with metabolic dysfunction-associated steatotic liver disease (MASLD) [9, 10]. MASLD is a chronic liver disease characterized by the association of hepatic steatosis with at least one cardiometabolic risk factor [11]. It includes simple liver steatosis, steatohepatitis (with various degrees of fibrosis), and hepatocellular carcinoma [11, 12]. In fact, MASLD is part of a multisystem disease and increases the long-term risk of fatal or non-fatal cardiovascular disease (CVD), independent of other risk factors [13, 14, 15]. The meta-analysis by Mantovani A *et al*. [15] showed that the cardiovascular risk increases significantly with more severe liver fibrosis (hazard ratio (HR): 2.50 [95% confidence interval (CI): 1.68–3.72]). Similar results were published previously by Targher G and colleagues, demonstrating that “more severe” MASLD (i.e., steatohepatitis with various amounts of fibrosis) was associated with an increased risk of fatal and non-fatal CVD events (random effect odds ratio (OR): 1.94 [95% CI: 1.17; 3.21]) [16].

We have recently reported that T2DM patients with MASLD and advanced liver fibrosis presented lower EF, cardiac hypertrophy and markers of diastolic dysfunction [17]. The study also demonstrated that more severe hepatic fibrosis was associated with progressively higher EATT. In fact, the work by Petta *et al*. [18] had also showed that EATT was associated with the severity of liver fibrosis in MASLD patients. However, there is scarce data regarding these correlations in patients with T2DM and MASLD. Furthermore, there are many unresolved questions regarding the complex interplay between MASLD and EAT, including the mechanisms underlying these associations or the value of EATT as a predictor of advanced liver disease. This work aimed at evaluating the association between EATT and liver fibrosis and steatosis in T2DM patients with MASLD.

## 2. Materials and Methods

### 2.1 Study Population

T2DM patients with MASLD were enrolled from July 2022 until July 2023 in the Outpatient Unit of the Emergency County Clinical Hospital of Târgu Mureș, Romania. The details regarding the materials and methods used, including study population, clinical, laboratory and echocardiographic evaluation, were published elsewhere [17, 19]. Briefly, subjects with T2DM were included if they were 30 years of age or older and had non-alcoholic fatty liver disease (NAFLD). NAFLD definition (liver steatosis/steatohepatitis in the absence of secondary causes of hepatic disease) was used at study entry as an inclusion criterion, but in June 2023 there was a change in definition and terminology (to MASLD), which was largely used thereafter [11]. All study patients fulfilled the proposed definition of MASLD, and we therefore have adopted the new terminology to characterize this study population. The study was approved by the Ethics Committees of the Emergency County Clinical Hospital of Târgu Mureș (nr. 8120/05.04.2022), County Clinical Hospital of Târgu Mureș (nr. 4873/24.05.2022), and George Emil Palade University of Medicine, Pharmacy, Science and Technology of Târgu Mureș (nr. 1806/22.06.2022). The informed consent was signed by all patients enrolled in the study.

### 2.2 Clinical and Laboratory Evaluation

Information regarding demographic and medical data (including personal history, current therapy, and lifestyle) was obtained through several questionnaires. Heart rate, blood pressure, pO2, as well as several anthropometric parameters (weight, height, waist and hip circumferences) were measured by standard methods. Additional anthropometric data was obtained by using an InnerScan BC-545N segmental body composition monitor (lot nr. 5210112, Tanita; Tokyo, Japan). The assessment of hepatic steatosis was performed by ultrasonographic (US) B-mode imaging using a Hitachi Arietta v70 system (Model P95DE, Mitsubishi Electric Corporation; Kyoto, Japan) [20].

Blood was drawn in fasting conditions on the same day. The complete blood count (CBC) was analyzed immediately afterwards on a 5-part differential automated hematology equipment (Mindray BC6200, India). Serum aliquots were stored at –80 °C for subsequent analyses of multiple parameters: metabolic panel (glycated hemoglobin (HbA1c), glucose, C-peptide, uric acid, total cholesterol, low-density lipoprotein (LDL) cholesterol, high-density lipoprotein (HDL) cholesterol, triglycerides), liver panel (albumin, aspartate aminotransferase (ASAT), alanine aminotransferase (ALAT), gamma glutamyl transpeptidase (GGT), direct bilirubin), creatinine, sex hormone-binding globulin (SHBG), ferritin, haptoglobin. The analysis of the biochemical parameters was performed on a Cobas Integra 400plus equipment (Roche Diagnostics; Mannheim, Germany), by using an immunoturbidimetric method (for HbA1c, albumin and haptoglobin), and a spectrophotometric method (for glucose, uric acid, lipid panel, creatinine, and liver panel). Reagents were obtained from Roche Diagnostics (Mannheim, Germany). The C-peptide, ferritin and SHBG were measured by a solid phase, two-site chemiluminescent immunometric assay on an Immulite 2000 XPI system (Siemens Healthcare Diagnostics; Erlangen, Germany). Reagents were obtained from Siemens Healthcare Diagnostics Products Ltd. (Llanberis, UK).

### 2.3 Echocardiographic Evaluation

The transthoracic two-dimensional echocardiographic assessment was performed within 2–3 weeks from the initial visit by an experienced cardiologist, blinded to the other aspects of the study, by using a VIVID9 XDClear equipment (GE HealthCare; GE Vingmed Ultrasound AS, Horten, Norway). The details regarding the cardiac US evaluation (performed by standard techniques in accordance with the recommendations of the ASE/EAC Guidelines) were published before [17, 21]. Here we report data related to the EATT, which was measured with the patient positioned in the left lateral decubitus. Measurements were taken perpendicularly on the free wall of the right ventricle from a parasternal long-axis view. EAT appeared as an echo-lucent space between the outer myocardial surface and the visceral layer of the pericardium. The measurement was performed at end-diastole, across three consecutive cardiac cycles, and the mean value was recorded in millimeters. EATT measurements were performed by a single experienced cardiologist, ensuring internal consistency.

### 2.4 Calculations

The body mass index (BMI) was calculated with the formula: weight/height^2^ (kg/m^2^), and body fat mass (BFM) (kg) was computed from weight and % body fat. For the estimated glomerular filtration rate (eGFR) the CKD-EPI 2021 formula was used [22]. The Homeostatic Model Assessment (HOMA) for Insulin Resistance (HOMA-IR) was calculated with the HOMA calculator version 2.2.3 [23].

Three inflammatory indexes were calculated from the CBC data: Neutrophil-to-Lymphocyte Ratio (NLR), Systemic Immune-Inflammation Index (SIII) (platelet count × neutrophil count/lymphocyte count), and Systemic Inflammatory Response Index (SIRI) (neutrophil count × monocyte count/lymphocyte count) [24].

Liver steatosis was confirmed by US in all subjects (as was a mandatory inclusion criterion, per NAFLD/MASLD definition). In addition, we have used several non-invasive indexes for liver steatosis evaluation/grading: Fatty liver index (FLI), Hepatic Steatosis Index (HSI) and Index of NASH (Non-alcoholic steatohepatitis) (ION), using following formulas: FLI = (e^0.953×loge (TG) + 0.139×BMI + 0.718×loge (GGT) + 0.053×waist – 15.745^)/(1 + e^0.953×loge (TG) + 0.139×BMI + 0.718×loge (GGT) + 0.053×waist –^^15.745^) × 100 (30 rules out and ≥60 rules in steatosis), HSI = 8 × (ALAT/ASAT) + BMI (kg/m^2^) (+2, if female; +2, if DM) (<30 rules out and >36 rules in steatosis), and ION = 1.33 × waist-to-hip ratio + 0.03 × triglycerides (mg/dL) + 0.18 × ALAT (U/L) + 8.53 × HOMA-IR – 13.93 (for men), and ION = 0.02 × triglycerides (mg/dL) + 0.24 × ALAT (U/L) + 9.61 × HOMA-IR – 13.99 (for women), respectively (a score <11 excludes steatosis, >22 indicates steatosis, while ION score >50 predicts NASH) [25, 26, 27]. Since all patients had US-confirmed liver steatosis, in order to identify subjects with more severe liver steatosis, a combination of FLI and HSI thresholds was used (as these are the most widely accepted steatosis indexes), and we defined the more severe hepatic steatosis group as having both FLI ≥60 and HSI >36, while the rest of the subjects were considered as having moderate steatosis.

Liver fibrosis was estimated by several validated non-invasive indexes: Fibrosis-4 (FIB-4) score, NAFLD-Fibrosis Score (NFS), and GGT to platelet ratio (GPR), by using following formulas: FIB-4 = age (years) × ASAT (U/L)/[platelet (10^9^/L) × ALAT^1/2^ (U/L)] (<1.3 rules out advanced fibrosis, a score >2.67 rules in advanced fibrosis (F ≥2), while values between 1.3–2.67 are considered indeterminate), NFS = –1.675 + 0.037 × age (years) + 0.094 × BMI (kg/m^2^) + 1.13 × impaired fasting glucose (IFG)/DM (yes = 1; no = 0) + 0.99 × ASAT/ALAT – 0.013 × platelet (×10^9^/L) – 0.66 × albumin (g/dL) (>0.676 indicate significant fibrosis (>F2), <–1.455 indicates no significant fibrosis, while values between –1.455 to 0.676 are undetermined), and GPR = (GGT (U/L)/ULN)/platelet count (10^9^/L) × 100 (where ULN is the upper normal limit for GGT) [28, 29, 30]. In order to better segregate the advanced fibrosis group (in the absence of liver histology), we empirically combined the three fibrosis indexes and divided the study population accordingly: with advanced liver fibrosis (FIB-4 >2.67 and NFS >–1.455 *or* NFS >0.676 and FIB-4 >1.3, *and* GPR ≥median value) and without advanced fibrosis (which comprised the rest of subjects not fulfilling either criterion; i.e., those with indeterminate risk or no risk of advanced fibrosis).

The left ventricular (LV) mass (LVM) was calculated with the formula: LVM (g) = 0.80 × [1.04 × (PWd (cm) + IVSd (cm) + LVDd (cm))^3^ – (LVDd (cm))^3^] + 0.6 (1.04 is the heart muscle density (g/cm^3^), PWd = LV posterior wall thickness at end-diastole; IVSd = interventricular septum thickness at end-diastole, LVDd = LV end-diastolic dimension) [21]. The LVM was indexed (LVMi) to the body surface area (calculated with the DuBois formula) [31].

### 2.5 Statistical Analysis

Data were analyzed using descriptive statistics and the normality of data was checked by using the Kolmogorov-Smirnov test. Results are presented as mean ± standard deviation (SD) for normally distributed data, and median (interquartile range (IQR)) for non-parametric data. Continuous variables were compared by using the unpaired *t* test (for normally distributed data), Welch corrected (if SD were significantly different) or Mann-Whitney test (for non-parametric data), while the categorical variables were analyzed by using the Fisher’s exact test, and ORs [95% CI] reported. The association between two variables was tested with Spearman’s test (for non-parametric data) or Pearson’s test (for data with Gaussian distribution), and results presented as r [95% CI]. The multiple regression analyses were applied for more than two sets of variables to test the independent associations between EATT and hepatic steatosis and fibrosis indexes or other significant variables. Additional sensitivity analyses were performed to identify the optimal EATT cut-off value that predicts advanced liver fibrosis by using receiver operating characteristics (ROC) analyses, and the Area Under the ROC Curve (AUROC) was calculated (with value of 1 indicating perfect performance). Statistical significance was set at *p* < 0.05. Statistical analysis was performed by using GraphPad InStat3 software (GraphPad Software, Boston, USA), and the IBM SPSS stat version 31.0.0.0 (IBM Corp., NY, USA) for additional analyses (i.e., ROC and multiple regression analyses).

## 3. Results

Data from 267 T2DM patients with MASLD who had a complete medical evaluation (including echocardiography) were analyzed. The characteristics of the overall study population were presented elsewhere [17]. The median EATT value in this study population was 7 mm, and therefore patients were divided into two groups (median-split): with EATT >7 mm (43.8%) and EATT ≤7 mm (56.2%), respectively. There were no significant differences between the two groups with regards to age (66.00 (9.0) years vs. 66.50 (10.0) years; *p* = 0.9994) or duration of diabetes (9.00 (6.5) years vs. 10.00 (5.0) years; *p = *0.0729). Instead, patients with higher EATT had higher body adiposity, GGT, ferritin, C-peptide and HOMA-IR, and lower SHBG values compared to the lower EATT group (Table 1).

**Table 1.** **Clinical and laboratory characteristics of study groups, according to the median EATT value**.

		EATT >7 mm	EATT ≤7 mm	*p* value
		(n = 117)	(n = 150)	
Clinical data				
	Sex (female/male) (no)	68/49	78/72	0.3251
	BMI (kg/m^2^)	35.35 ± 5.60	32.46 (6.02)	0.0008
	Waist circumference (cm)	114.58 ± 11.41	109.59 ± 11.43	0.0005
	Hip circumference (cm)	111.45 ± 10.53	105.95 (12.83)	0.0058
	% body fat	38.07 ± 7.57	35.75 (11.22)	0.0075
	Body fat mass (kg)	36.17 ± 10.47	30.30 (12.05)	0.0004
	Alcohol intake (g/day)	0.40 (1.72)	0.52 (3.76)	0.1500
	Smoking (yes/no) (no)	16/101	12/138	0.1600
	Systolic BP (mmHg)	134.86 ± 16.19	135.0 (20.0)	0.9262
	Diastolic BP (mmHg)	81.0 (12.5)	80.0 (12.5)	0.6102
	Heart rate (beats/min)	73.0 (14.0)	74.0 (12.25)	0.7823
	pO2 (%)	97.0 (2.0)	98.0 (1.0)	0.0032
Laboratory data				
	Fasting blood glucose (mg/dL)	142.28 ± 27.80	136.93 (30.52)	0.8756
	HbA1c (%)	6.80 (1.0)	6.90 (0.82)	0.9987
	Total cholesterol (mg/dL)	153.20 (48.78)	155.16 (44.64)	0.6290
	HDL cholesterol (mg/dL)	43.51 (11.88)	44.11 (12.25)	0.7658
	LDL cholesterol (mg/dL)	80.85 (43.08)	83.31 (39.29)	0.6160
	Triglycerides (mg/dL)	154.63 (90.97)	150.94 (84.66)	0.7033
	C-peptide (ng/mL)	3.33 (2.18)	3.01 (1.82)	0.0262
	HOMA-IR	2.89 (1.78)	2.61 (1.67)	0.0335
	Uric acid (mg/dL)	5.87 (2.01)	5.75 ± 1.43	0.0893
	Albumin (g/dL)	4.62 (0.25)	4.66 ± 0.24	0.2846
	ASAT (U/L)	22.00 (13.15)	19.83 (9.11)	0.0766
	ALAT (U/L)	19.26 (18.35)	17.73 (12.91)	0.4743
	GGT (U/L)	31.19 (33.57)	27.03 (21.87)	0.0164
	Direct bilirubin (mg/dL)	0.20 (0.10)	0.20 (0.10)	0.6686
	Ferritin (ng/mL)	113.00 (134.05)	79.55 (118.85)	0.0472
	Haptoglobin (g/L)	1.69 ± 0.59	1.68 ± 0.61	0.8938
	eGFR (mL/min/1.73 m^2^)	88.30 (23.77)	91.68 (21.91)	0.1130
	SHBG (nmol/L)	31.80 (18.50)	35.65 (19.8)	0.0343
	NLR	2.027 (1.14)	1.846 (0.97)	0.2929
	SIII	459.29 (315.29)	442.04 (285.32)	0.5734
	SIRI	0.938 (0.62)	0.867 (0.67)	0.3647

EATT, epicardial adipose tissue thickness; no, number; BMI, body mass index; BP, blood pressure; HbA1c, glycated hemoglobin; HDL, high-density lipoprotein; LDL, low-density lipoprotein; HOMA-IR, Homeostatic Model Assessment for Insulin Resistance; ASAT, aspartate aminotransferase; ALAT, alanine aminotransferase; GGT, gamma glutamyl transpeptidase; eGFR, estimated glomerular filtration rate; SHBG, sex hormone-binding globulin; NLR, Neutrophil-to-Lymphocyte Ratio; SIII, Systemic Immune-Inflammation Index; SIRI, Systemic Inflammatory Response Index; data is presented as mean ± SD if normally distributed, and median (IQR) if non-normally distributed, respectively.

Patients in the higher EATT category had a slightly lower EF (48.89 ± 4.91 [50.0 (3.0)]% vs 51.65 ± 5.20 [52.0 (6.0)]%; *p* < 0.0001) and higher LVMi (119.28 ± 26.27 g/m^2^ vs 110.74 ± 24.58 [108.57 (26.74)] g/m^2^; *p* = 0.0107).

### EATT and Liver Steatosis and Fibrosis

**Fig. 1.**
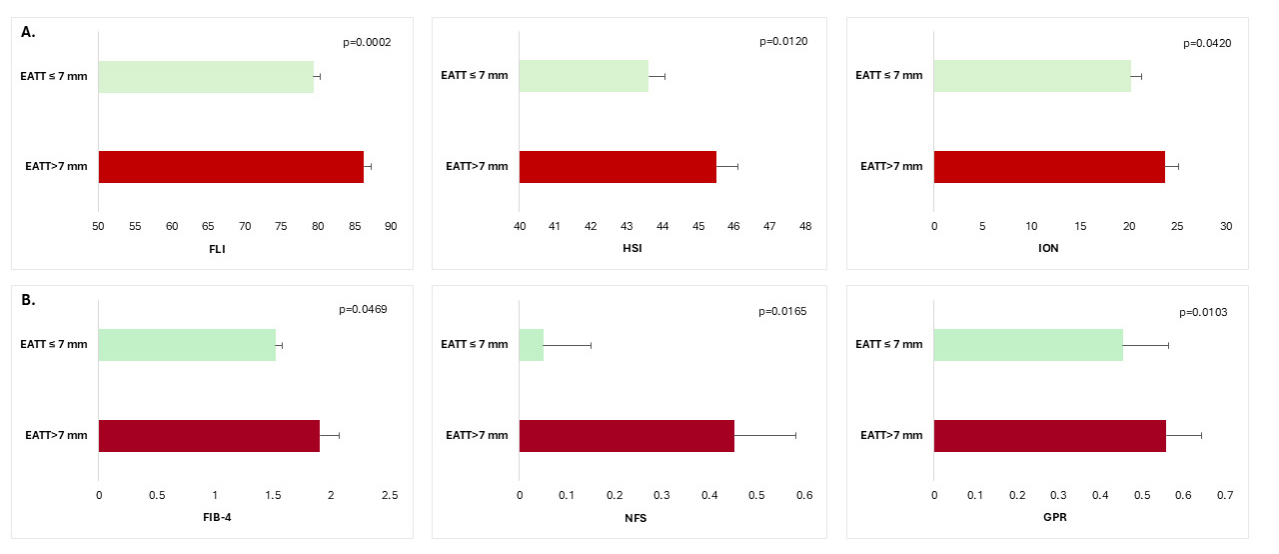
**Non-invasive biomarkers of hepatic steatosis (A) and hepatic fibrosis (B) according to EAT thickness in patients with T2DM and MASLD**. FLI, fatty liver index; HSI, hepatic steatosis index; ION, index of NASH (Non-alcoholic steatohepatitis); FIB-4, Fibrosis-4 score; NFS, NAFLD-Fibrosis score; GPR, gamma glutamyl transpeptidase to platelet ratio; T2DM, type 2 diabetes mellitus; MASLD, metabolic dysfunction-associated steatotic liver disease; EAT, epicardial adipose tissue; data are means ± standard error of means (SEM).

Patients with an EATT higher than 7 mm presented higher values of hepatic steatosis indexes (FLI: 86.29 ± 15.89 [93.40 (18.15)] vs 79.35 ± 18.65 [85.00 (26.55)], *p* = 0.0002; HSI: 45.51 ± 6.41 vs 43.61 ± 5.70 [42.88 (7.2)], *p* = 0.0120; ION: 23.69 ± 14.98 [21.56 (19.33)] vs 20.16 ± 13.75 [19.02 (18.77)], *p* = 0.0420) and hepatic fibrosis indexes (FIB-4: 1.90 ± 1.83 [1.52 (0.99)] vs 1.52 ± 0.74 [1.31 (0.64)], *p* = 0.0469 (one outlier value excluded); NFS: 0.45 ± 1.41 vs 0.050 ± 1.20 [0.027 (1.31)], *p* = 0.0165; GPR: 0.56 ± 0.91 [0.31 (0.34)] vs 0.45 ± 1.37 [0.25 (0.25)], *p* = 0.0103) compared with patients with an EATT <7 mm (Fig. 1A,B).

**Fig. 2.**
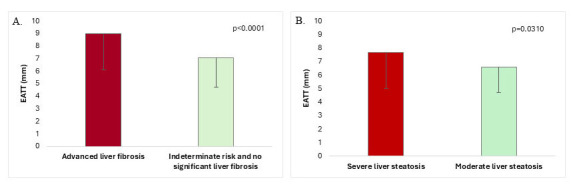
**EATT in T2DM-MASLD patients with and without advanced liver fibrosis (A) and EATT in T2DM-MASLD patients with severe vs moderate liver steatosis (B) (data is means ± SD)**. EATT, epicardial adipose tissue thickness; T2DM, type 2 diabetes mellitus; MASLD, metabolic dysfunction-associated steatotic liver disease.

The EATT was significantly higher in the advanced liver fibrosis group (defined by the combination of the three fibrosis indexes) (n = 60) (8.97 ± 2.88 [8.0 (3.0)] mm vs 7.09 ± 2.38 [7.0 (4.0)] mm; *p* < 0.0001) (Fig. 2A). Moreover, there was a significant difference between the percentage of patients with EATT >7 mm in the two groups (68.3% vs 36.7%; OR = 3.72 [95% CI: 2.02; 6.87]; *p* < 0.0001).

To verify the approach and results, further ROC analyses were performed. For the FIB-4, the AUROC was 0.598 ([95% CI: 0.489; 0.707], *p* = 0.079), for NFS the AUROC was 0.627 ([95% CI: 0.553; 0.700], *p* = 0.001), for GPR was 0.619 ([95% CI: 0.551; 0.686], *p* = 0.001), while for the three fibrosis indexes combined the AUROC was 0.696 ([95% CI: 0.623; 0.769], *p* = 0.000). The EATT value of 7.2 mm had a sensitivity of 0.683 and 1-specificity of 0.367 (Youden’s index = 0.316), while the EATT value of 7.7 mm had the same sensitivity and the 1-specificity of 0.362 (Youden’s index = 0.321) (for all the other calculated cut-offs, the Youden’s index was lower). Of note, in our database only one subject had an EATT value of 7.4 mm, the rest had either ≤7 mm or ≥8 mm, suggesting that the median-split grouping based on EATT values of ≤7 mm/>7 mm is reasonable, since choosing the threshold of 7.7 mm would only re-classify one subject.

Patients with more severe hepatic steatosis had higher EATT (7.69 ± 2.70 [7.0 (3.0)] mm vs. 6.61 ± 1.88 [7.0 (3.0)] mm; *p* = 0.0310) (Fig. 2B). More patients had an EATT value >7 mm in the more severe liver steatosis group (46.88% vs 27.91%; OR = 2.28 [95% CI: 1.11; 4.67]; *p* = 0.0285). Similar ROC analyses were performed for liver steatosis indexes. For FLI the AUROC was 0.603 ([95% CI: 0.508; 0.698], *p* = 0.049), for HSI was 0.615 ([95% CI: 0.489; 0.739], *p* = 0.064), for ION was 0.562 ([95% CI: 0.493; 0.631], *p* = 0.035), while the combination of FLI and HSI it was 0.604 ([95% CI: 0.519; 0.689]), *p* = 0.043.

In the bivariate analyses EATT significantly correlated with markers of body adiposity (waist, hip circumference, BMI, % body fat), non-invasive indexes of liver steatosis (FLI, HSI) and fibrosis (NFS, GPR), ASAT, GGT, diabetes duration, and pO2 (Table 2). For FIB-4, a weak positive correlation was noted, although statistical significance was not quite reached (r = 0.12 [95% CI: –0.004; 0.24], *p* = 0.0504). For the rest of the variables no significant associations were observed (including ION (r = 0.12 [95% CI: –0.01; 0.24], *p* = 0.0598) and C-peptide (r = 0.12 [95% CI: –0.004; 0.24], *p* = 0.0513), for which the non-significance was borderline though).

**Table 2.** **Parameters and indexes significantly correlated with EATT in bivariate analyses**.

	Correlation coefficient r [95% CI]	*p* value
Waist circumference	0.23 [0.11; 0.34]	0.0002
Hip circumference	0.18 [0.06; 0.29]	0.0036
BMI	0.20 [0.08; 0.32]	0.0009
% body fat	0.13 [0.01; 0.25]	0.0358
Body fat mass (kg)	0.21 [0.09; 0.32]	0.0006
NFS	0.13 [0.01; 0.25]	0.0312
GPR	0.18 [0.06; 0.30]	0.0024
FLI	0.24 [0.12; 0.35]	<0.0001
HSI	0.16 [0.04; 0.28]	0.0086
ASAT	0.13 [0.01; 0.25]	0.0326
GGT	0.20 [0.08; 0.31]	0.0012
Ferritin	0.19 [0.07; 0.31]	0.0019
Diabetes duration	–0.12 [–0.24; 0.001]	0.0443
pO2	–0.19 [–0.30; –0.07]	0.0020

BMI, body mass index; NFS, NAFLD-Fibrosis Score; GPR, GGT to platelet ratio; FLI, Fatty liver index; HSI, Hepatic Steatosis Index; ASAT, aspartate aminotransferase; GGT, gamma glutamyl transpeptidase; data are coefficient of correlation r [95% confidence interval].

Three sets of multiple regression analyses were performed to identify the parameters independently correlated with EATT. In model 1, markers of liver fibrosis (FIB-4 and NFS respectively), diabetes duration, sex, pO2, GGT, ferritin, C-peptide and SHBG were used as independent variables, in model 2 BFM was added (as these parameters were identified as significantly correlated with EATT in the bivariate analyses), while in model 3 a full adjustment was done with waist, HbA1c and HOMA-IR (instead of C-peptide) added as independent variables. The same was done for the markers of liver steatosis (FLI and HSI, respectively), except that ASAT was used instead of GGT. EATT correlated significantly with FIB-4 in all models, while NFS was independently correlated only in model 1, but after adjustment for body adiposity the correlation became non-significant. In contrast, in both fully adjusted models, serum ferritin was significantly correlated with EATT (Table 3). EATT was positively correlated with both liver steatosis indexes, but after adjustments for body adiposity the correlations became non-significant (Table 3). Instead, serum ferritin was positively correlated with EATT in the fully adjusted models.

**Table 3.** **Correlations between EATT and indexes of liver fibrosis (a) and liver steatosis (b), in multiple regression analyses**.

			Independent variable	Standardized coefficient β [SE]	95% CI	t ratio
a. Hepatic fibrosis						
	Assessed by FIB-4					
		Model 1	FIB-4	0.165 [0.123]*	0.077; 0.562	2.598
		R^2^ = 0.082; *p* = 0.005				
		Model 2	FIB-4	0.165 [0.122]**	0.079; 0.561	2.615
		R^2^ = 0.096; *p* = 0.002	BFM	0.141 [0.018]*	0.001; 0.073	2.014
		Model 3	FIB-4	0.169 [0.122]**	0.087; 0.566	2.687
		R^2^ = 0.122; *p * < 0.001	Ferritin	0.164 [0.001]*	0.001; 0.006	2.353
	Assessed by NFS					
		Model 1	NFS	0.131 [0.127]*	0.012; 0.511	2.067
		R^2^ = 0.075; *p* = 0.009				
		Model 2	NFS	0.101 [0.132]	–0.057; 0.462	1.536
		R^2^ = 0.083; *p* = 0.007				
		Model 3	NFS	0.093 [0.132]	–0.074; 0.447	1.407
		R^2^ = 0.108; *p* = 0.003	Ferritin	0.166 [0.001]*	0.001; 0.006	2.365
b. Hepatic steatosis						
	Assessed by FLI					
		Model 1	FLI	0.193 [0.10]**	0.008; 0.048	2.769
		R^2^ = 0.074; *p* = 0.010				
		Model 2	FLI	0.147 [0.013]	–0.004; 0.047	1.655
		R^2^ = 0.077; *p* = 0.014				
		Model 3	FLI	0.110 [0.015]	–0.012; 0.045	1.115
		R^2^ = 0.100; *p* = 0.007	Ferritin	0.152 [0.001]*	0.000; 0.006	2.083
	Assessed by HSI					
		Model 1	HSI	0.148 [0.30]*	0.005; 0.122	2.131
		R^2^ = 0.063; *p* = 0.030				
		Model 2	HSI	0.057 [0.045]	–0.065; 0.114	0.542
		R^2^ = 0.068; *p* = 0.032				
		Model 3	HSI	0.014 [0.046]	–0.085; 0.097	0.129
		R^2^ = 0.096; *p* = 0.011	Ferritin	0.159 [0.001]*	0.000; 0.006	2.177

In model 1 the independent variables were FIB-4 or NFS (for liver fibrosis) and FLI or HSI (for liver steatosis) and diabetes duration, sex, pO2, GGT (for liver fibrosis equations) or ASAT (for liver steatosis equations), ferritin, C-peptide and SHBG; in model 2 the body fat mass (BFM) was added to above mentioned parameters and indexes; model 3 further adjusted for waist, HbA1c and HOMA-IR (without C-peptide); *p < 0.05; **p < 0.01. SE, standard error; FIB-4, Fibrosis-4; NFS, NAFLD-Fibrosis Score; BFM, body fat mass; FLI, Fatty liver index; HSI, Hepatic Steatosis Index.

## 4. Discussions

The ectopic fat accumulation is now recognized as a major risk factor for the development of cardio-metabolic diseases, through local and systemic effects [32]. EAT is a unique, quantifiable visceral fat depot that was previously suggested to predict hepatic steatosis in obese individuals [33]. The relationship between EAT and liver steatosis was previously evaluated, but less studies investigated the association with liver fibrosis [10]. Moreover, there is very limited data in the literature regarding this association in T2DM subjects with MASLD. The present study is among the first to explore the relationship between EAT and liver health in these individuals, and our findings indicate that increased EATT (>7 mm) is associated with adverse metabolic and cardiovascular parameters, more severe liver steatosis and advanced liver fibrosis. Hence, more severe MASLD-associated liver steatosis in the context of T2DM was correlated with higher EATT (an association apparently mediated by body adiposity), while markers of advanced fibrosis were independently correlated with EATT, highlighting the cardio-hepatic interrelationship in the context of metabolic diseases, with higher ectopic fat accumulation being strongly linked to more advanced liver disease. In a study of 100 patients with T2DM, Brouha *et al*. [34] reported previously that EAT volume quantified by cardiac computed tomography (CT) was increased in subjects with NAFLD, and it correlated independently with liver steatosis and fibrosis.

A key finding of this study was the significant association between EATT and liver fibrosis severity in T2DM patients with MASLD. Patients with markers of advanced fibrosis exhibited higher EATT values compared to those without severe fibrosis (8.97 ± 2.88 vs. 7.09 ± 2.38 mm), suggesting a potential link between increased epicardial fat and progressive liver fibrosis, and highlighting the potential role of EATT as a non-invasive marker for assessing liver disease severity. Like our results, the study by Petta *et al*. [18] has reported that subjects with biopsy-proven NAFLD had higher EATT in the presence of severe vs. milder liver fibrosis (8.5 ± 3.0 vs. 7.2 ± 2.3 mm; *p* = 0.006). Notably, in our study the stratification of patients based on fibrosis scores revealed that a considerably higher proportion of patients in the advanced fibrosis group had EATT values exceeding 7 mm (68.3% vs. 36.7%), with an almost 4-fold increased OR compared with the rest of the study subjects (with no or indeterminate risk of advanced fibrosis). T2DM patients with MASLD and more severe liver steatosis also had higher EATT values. Thus, EATT higher than 7 mm (and certainly higher than 7.7 mm) seem to be associated with more severe liver fibrosis and might help identify individuals at risk of advanced liver disease, but histological studies need to confirm or identify the best EATT cut-off value.

Furthermore, EATT positively correlated with non-invasive indices of liver fibrosis and steatosis, supporting its potential role as a biomarker for liver disease. The correlation with liver steatosis appeared mediated by body adiposity, while the association with liver fibrosis (FIB-4) was independent of other factors. The lack of significant correlation with NFS after body adiposity adjustment (as opposed to FIB-4) is not entirely clear, but it might be explained by differences in the parameters used for the calculations (i.e., NFS formula included BMI, a body adiposity marker). The same might be true for the liver steatosis indices. Although it is well known that adipocytes (mainly visceral) significantly contribute to hepatic steatosis through the release of free fatty acids, pro-inflammatory cytokines/adipokines, and other mechanisms, we cannot clearly differentiate here the role of body fat distribution (total vs. visceral/EAT) in causing liver steatosis [35, 36].

These observations underscore the interconnection between metabolic dysfunction, liver health, and cardiovascular risk, which is also supported by the study of Yilmaz *et al*. [37], that showed a complex interplay between EATT, EAT-related adipokines, liver histology and coronary blood flow in subjects with NAFLD. This is further substantiated by the work of Turan [38] which indicated that NFS was correlated with EATT and higher cardiovascular risk, and of Liu *et al*. [39] reporting that the increase in EATT was associated with more severe liver steatosis, fibrosis and CVD. A relatively small prospective study of 88 adults (46 with obesity and 42 healthy controls) confirmed the positive correlations between EAT and liver fat assessed by proton density fat fraction (PDFF), but not between EAT and liver stiffness [40]. However, only six subjects in this cohort presented liver fibrosis (assessed by magnetic resonance elastography) [40].

The normal value for EATT is not well defined, especially in subjects with T2DM and obesity, as some studies suggested a threshold of 5 mm, others of 7 mm or higher [8, 41, 42, 43, 44]. The study by Iacobellis *et al*. [43] reported a US-measured median EATT of 6.7–7 mm in individuals with overweight and class 1 and 2 obesity, and 8.9 mm in class 3 obesity (compared to a value of 4 mm in normal-weight individuals). In our study, patients with EATT >7 mm demonstrated significantly higher body adiposity (BMI, waist circumference, BFM) than those with lower EATT. Additionally, patients in the higher EATT group exhibited elevated levels of GGT, ferritin, and C-peptide, along with increased HOMA-IR, indicative of hepatic metabolic stress and systemic insulin resistance. These findings support prior evidence that epicardial fat strongly correlates with general adiposity and metabolic dysfunction, and align with existing literature that highlights the role of visceral fat, including EAT, as an essential contributor to metabolic syndrome and associated complications in T2DM patients [43, 45, 46].

Conversely, we have found that SHBG concentrations were lower in patients with elevated EATT. Similarly, the study by Aydogdu *et al*. [47] has shown a negative correlation between EATT and SHBG levels in women with polycystic ovary syndrome, and we have previously reported a negative correlation with LVMi and left atrium diameter. These results suggest a possible role of SHBG in metabolic dysregulation and cardiac health, but more in-depth research is required to fully elucidate the potential underlying pathophysiologic mechanisms.

Moreover, in all fully adjusted models of the multiple regression analyses ferritin was consistently correlated with EATT, suggesting that it might be a mediator between liver and cardiometabolic health. Serum ferritin was previously shown to be positively correlated with body adiposity, visceral adipose tissue, and EAT, and to be inversely associated with adiponectin levels in subjects with T2DM and obesity [48, 49, 50]. A causal relationship was suggested, as iron downregulated adiponectin transcription, and this was accompanied by increased insulin resistance [50]. Preclinical data also indicated that adipsin (a pericardial adipose tissue-derived adipokine) upregulated levels of ferritin heavy chain after myocardial infarction, suggesting a crosstalk between adipokines/adipocyte metabolism and iron metabolism [51]. On the other hand, serum ferritin was associated with liver inflammation and fibrosis in NAFLD patients, and hyperferritinemia was reported to be an independent predictor of MASLD-associated fibrosis in subjects with T2DM [52, 53]. Serum ferritin is a known inflammation marker, but it had been argued that it correlates with markers of cell stress and damage as well [54]. Thus, ferritin might mediate the link between visceral adipose tissue, insulin resistance and MASLD fibrosis, but further studies are needed to confirm this. The mechanisms behind EATT-liver fibrosis relationship are not entirely clear, but EATT is a highly active visceral adipose tissue depot, which releases bioactive factors, including fatty acids, proinflammatory and profibrotic factors, which may act in a paracrine or even endocrine fashion, thus playing an important role in the cardio-hepatic link [55, 56, 57].

The observed changes in echocardiographic parameters (i.e., lower EF and higher LVMi in patients with higher EATT), shed light on the cardiac implications of increased ectopic fat accumulation. These findings are consistent with studies linking visceral fat and EATT to adverse cardiovascular outcomes, underscoring the need for targeted interventions focusing on weight management in clinical practice [4, 5, 6, 58].

Overall, the findings of this study suggest that monitoring EATT could be a valuable clinical tool in managing patients with T2DM and MASLD. Identification of an increased US-measured EATT should prompt investigation of liver fibrosis status, but larger scale studies are needed to better define the significant cut-off value of EATT that would direct clinicians to further explore the liver health.

This study has several limitations. First, we could not use liver biopsy (the gold-standard method) to define steatosis or fibrosis, but instead several validated and accepted non-invasive indexes were employed. In fact, FIB-4 is the index recommended by guidelines for first-step screening for advanced liver fibrosis in individuals with T2DM [59, 60]. We have used three fibrosis biomarkers to ensure a proper selection of patients with advanced liver fibrosis, since none of them are perfect in predicting liver fibrosis [61]. Subsequent ROC analyses indicated that the AUROC was highest when this approach was used. Of course, this approach would need validation in future histology studies, as a potential misclassification of (advanced) fibrosis in the absence of histological evaluation is a possibility. In addition, similar findings were reported by the liver biopsy study of Petta *et al*. [18], which reassured the validity of our results. Secondly, we do not have longitudinal data to evaluate if the EATT changes would correlate with changes in liver steatosis or fibrosis. Further studies should explore this aspect, as well as the potential pathogenetic mechanisms linking EAT and liver fibrosis, and this work lays the ground for it. Thirdly, we did not have the possibility to use more advanced cardiac imaging methods (e.g., magnetic resonance or CT) for a more accurate quantification of EATT and EAT volume, yet echocardiography provides a reasonable accuracy for EATT measurement (even if it relies to some degree on the experience of the US operator), is not expansive or invasive, and is largely available in clinical practice. However, the intra-observer variability was not assessed in this study, limiting insights into reproducibility. Future studies should involve multiple observers and report metrics such as intra-class correlation coefficients (ICCs) to strengthen methodological reliability. Finally, the single-center design of this study precludes the extrapolation of the results in the general population, and certainly more studies in patients with different demographic and medical backgrounds are needed.

## 5. Conclusion

T2DM patients with MASLD and markers of advanced liver fibrosis have higher EATT, which was independently associated with liver fibrosis.

## Availability of Data and Materials

The datasets used and analyzed during the current study are available from the corresponding author on reasonable request.
